# Supplementary material for: The exon junction complex regulates the splicing of cell polarity gene dlg1 to control Wingless signaling in development
Source: eLife. 2016 Aug 18;5:e17200. doi: 10.7554/eLife.17200 (PMC5008907; doi:10.7554/eLife.17200)
Supplement: Supplementary file 1. — DOI: http://dx.doi.org/10.7554/eLife.17200.028 [file elife-17200-supp1.docx]

**Supplementary file 1. Fly genetics information and Primers.**

**Genetic crosses for Figures 1-5 and figure supplements are listed below:**

For Figure 1A, C-F, Figure 2A-F, Figure 3A, B, Figure 3-figure supplement 1B, C, E, F, Figure 4A, B, Figure 4-figure supplement 6G-J: *hs-flp^122^*; FRT42D, *M(2)58F*, *ubi*-*gfp*/CyO, P(*y+*) x FRT42D*, tsu^Δ18^*/CyO.

For Figure 1B, Figure 1-figure supplement 2A-F, Figure 4-figure supplement 6C-F: *hs-flp^122^*; FRT42D, *M(2)58F*, *ubi*-*gfp*/CyO, P(*y+*) x FRT42D*, mago^93D^*/CyO.

For Figure 1G-H: *hs-flp^122^*; FRT42D, *M(2)58F*, *ubi*-*gfp* /CyO, P(*y+*) x *fz3*-*lacZ*/+, FRT42D*, tsu^Δ18^*/CyO.

For Figure 2G-I: Sco/CyO, *wg-lacZ*; *dpp-*Gal4, UAS-*mCD8-gfp*/TM6B x UAS-*tsu RNAi* (Bloomington #28955)

For Figure 2J-K: *NRT-HA-wg*/Cyo; *hh-*Gal4, *dpp-*Gal4, UAS-*mCD8-gfp/*TM6B x UAS-*tsu RNAi* (Bloomington #28955)

For Figure 3C, D: *hs-flp^122^*; FRT42D, *M(2)58F*, *ubi*-*gfp*/CyO, P(*y+*) x FRT42D*, mago^69B^*/CyO.

For Figure 3E: *vg^BE^-*Gal4, UAS-*mCD8-gfp*, UAS-*tsu RNAi* (VDRC #107385) x UAS-*lacZ*

For Figure 3F: *vg^BE^-*Gal4, UAS-*mCD8-gfp*, UAS-*tsu RNAi* (VDRC #107385) x UAS-*fz2*

For Figure 3G: *vg^BE^-*Gal4, UAS-*mCD8-gfp*, UAS-*tsu RNAi* (VDRC #107385) x UAS-*arr*

For Figure 3H: *vg^BE^-*Gal4, UAS-*mCD8-gfp*, UAS-*tsu RNAi* (VDRC #107385) x UAS-*dsh*

For Figure 3J, Figure 3-figure supplement 3: *T80-*Gal4, UAS-*mCD8-rfp* x UAS-*tsu RNAi* (VDRC #107385) and *T80-*Gal4, UAS-*mCD8-rfp* x UAS-*lacZ*

For Figure 4C, D, Figure 4-figure supplement 3A, B: *ubi*-*gfp*, FRT101/FM7; *esg-*Gal4, UAS-*flp* x *dlg1^14^*, FRT101;

For Figure 5C, D: *ap-*Gal4, UAS-*mCD8-gfp*/CyO; *ts*-Gal80 x UAS-*dlg1 RNAi* (Bloomington #35772). The flies were maintained at 18ºC for 10 days before shifted to 29ºC at early third instar larval stage for 12 hours. Wandering third instar larvae imaginal discs were then dissected for immunostaining.

For Figure 5E, F: *ap-*Gal4/CyO; UAS-*dlg1*-*gfp*

For Figure 5H-M: *dsh-gfp/+*; *dpp-*Gal4/+ x UAS-*Ubpy RNAi* (VDRC #107623)

For Figure 1-figure supplement 1A, B: *vg^BE^-*Gal4, UAS-*mCD8-gfp* x UAS-*gfp*

For Figure 1-figure supplement 1E: *vg^BE^-*Gal4, UAS-*mCD8-gfp* x UAS-*tsu RNAi* (VDRC #107385)

For Figure 1-figure supplement 1F: *vg^BE^-*Gal4, UAS-*mCD8-gfp* x UAS-*mago RNAi* (VDRC #28132)

For Figure 1-figure supplement 1G: *vg^BE^-*Gal4, UAS-*mCD8-gfp* x UAS-*eIF4AIII RNAi* (VDRC #108580)

For Figure 1-figure supplement 1H-J: *Dll-lacZ*/CyO, *hh-*Gal4/TM6B x UAS-*tsu RNAi* (Bloomington #28955)

For Figure 1-figure supplement 3: *hs-flp^122^*; FRT82B, *ubi*-*gfp* x FRT82B*, btz^2^*/TM6B

For Figure 4-figure supplement 1A: *da-*Gal4 x UAS-*gfp* and *da-*Gal4 x UAS-*tsu RNAi* (VDRC #107385)

For Figure 4-figure supplement 3C-D: *hh-*Gal4, UAS-*mCD8-gfp*/TM6B x UAS-*dlg1 RNAi* (Bloomington #35772)

For Figure 4-figure supplement 3E, E’: *vg^BE^-*Gal4, UAS-*mCD8-gfp*; UAS-*dlg1 RNAi* (Bloomington #35772) x UAS-*lacZ*

For Figure 4-figure supplement 3F, F’: *vg^BE^-*Gal4, UAS-*mCD8-gfp*; UAS-*dlg1 RNAi* (Bloomington #35772) x UAS-*dlg1*

For Figure 4-figure supplement 3G, G’: *vg^BE^-*Gal4, UAS-*mCD8-gfp*; UAS-*dlg1 RNAi* (Bloomington #35772) x UAS-*dsh*

For Figure 4-figure supplement 3H, H’: *vg^BE^-*Gal4, UAS-*mCD8-gfp*; UAS-*tsu RNAi* (VDRC #107385) x UAS-*dlg1*

For Figure 4-figure supplement 4B, C, E, F, H, I: *hh-*Gal4, UAS-*mCD8-gfp*/TM6B x UAS-*Rnps1 RNAi* (Bloomington #36580)

For Figure 5-figure supplement 1A, B, Figure 5-figure supplement 2A-D: *hs-flp^122^*; *ubi*-*gfp*, FRT40A/CyO x *l(2)gl^27S3^*, FRT40A/CyO

For Figure 5-figure supplement 1C, D, Figure 5-figure supplement 2E-H: *ubi*-*gfp*, *hs-flp^122^*, FRT9-2/y x *baz^4^*, FRT9-2/CyO

For Figure 5-figure supplement 1E, F, Figure 5-figure supplement 2I-L: *ubi*-*gfp*, *hs-flp^122^*, FRT19A/y x *cdc42^4^*, FRT19A/CyO

**Primers**

*Primers used in molecular cloning are listed below:*

*dsh-Myc*: 5’-ctgcagtaaagtgcaagttaaagtg-3’ and 5’-tgtccaattatgtcacaccacaga-3’,

*HA-dlg1*: 5’-gcggccgcatgtacccttacgacgttcctgattacgctagcctcacaacgaggaaaaagaagcg-3’ and 5’-tctagattatagagattccttggaaggtac-3’,

*HA-dlg1-RB*: 5’-gcggccgcatgtacccttacgacgttcctgattacgctagcctcccagtgaaaaagcaagaagc-3’ and 5’-tctagattatagagattccttggaaggtac-3’,

*dvl1-HA*: 5’-gccgaattcatggcggagaccaagattat-3’ and

5’-gcctctagattagaggctagcgtaatcaggaacgtcgtaagggtacatgatgtccacgaagaact-3’,

*dvl2-HA*: 5’-gccgaattcatggcgggtagcagcact-3’ and

5’-gcctctagattagaggctagcgtaatcaggaacgtcgtaagggtacataacatccacaaagaactcg-3’,

*dvl3-HA*: 5’-gccgaattcatgggcgagaccaagatc-3’ and

5’-gcctctagattagaggctagcgtaatcaggaacgtcgtaagggtacatcacatccacaaagaactc-3’,

*dlg2-Flag*: 5’-gcctctagagccaccatgggtatctttaagagcag-3’ and

5’-gcctctagataacttttcctttgagggaatcc-3’,

*dlg3-Flag*: 5’-gccggtacccacaagcaccagcactgctg-3’ and

5’-attgcggccgcttagagtttttcaggggatgg-3’,

*dlg2KKAA-Flag*: 5’-ttcaatgacaagcgtgcagctagcttcatcttttc-3’ and

5’-gaaaagatgaagctagctgcacgcttgtcattgaa-3’,

*dlg2 SFI-NL-Flag*: 5’-gacaagcgtaaaaagaacttgttttcacgaaaattc-3’ and

5’-gaattttcgtgaaaacaagttctttttacgcttgtc-3’.

*Primers used to generate dsRNAs are listed below:*

*tsu*: 5’-taatacgactcactataggggagaggcgatccacagctac-3’ and

5’-taatacgactcactatagggagtcgtttatctgcgacgct-3’,

*mago*: 5’-taatacgactcactatagggcaagctgcggtacgccaacaa-3’ and

5’-taatacgactcactatagggcaggccgatgagtgagaagacca-3’,

*dlg1*: 5’-taatacgactcactatagggtagggacacaccacccgacccaag-3’ and

5’-taatacgactcactatagggccattttaatcgcccgctcgt-3’,

*Gal80*: 5’-taatacgactcactatagggattaagcggccgcaacatggactacaacaagagatc-3’ and

5’-taatacgactcactataggggcgtgtctagattataaactataatgcgagatattg-3’.

*Primers used in quantitative real-time PCR are listed below:*

*α-Tub84B*: 5’-ggcaaggagatcgtcgatctgg-3’ and 5’-gacgctccatcagcagcgag-3’,

*tsu*: 5’-tgtgttggacattgacaa-3’ and 5’-cttcgccttttccttcag-3’,

*rl*: 5’-atatcgccctttgaacac-3’ and 5’-ctctcatttggtctatgctat-3’,

*cno*: 5’-agccctaacgatccagttg-3’ and 5’-catctgagagtttgccacaat-3’,

*dbo*: 5’-aggatttctctacgcaatt-3’ and 5’-ctacccatttgttgtgtct-3’,

*kel*: 5’-cttggttacagacgcaact-3’ and 5’-aagtggagtacaacgaagac-3’,

*l(2)gl*: 5’-tggtgcgatgtttactatttg-3’ and 5’-agactgtactggcatttgt-3’,

*Nek2*: 5’-gaagggcaggaaatacgat-3’ and 5’-gtcttagggcacacatctc-3’,

*RhoGEF2*: 5’-aggaaaccctatggatgac-3’ and 5’-ctgaatgcttgactcttctg-3’,

*sano*: 5’-gaagagcaactacaactt-3’ and 5’-aatactccattggaatctg-3’,

*yki*: 5’-aagccaagaccaatgatg-3’ and 5’-tggcgatattggattctg-3’,

*dlg1*: 5’-atactttacgggcgttgt-3’ and 5’-cgactgggaccaaatcat-3’,

*dsh*: 5’-tggctaaagataacgattcc-3’ and 5’-ctgaacatcctccacattc-3’.

*Primers used in RT-PCR are listed below:*

*α-Tub84B*: 5’-cgtttgtcaagcctcatagccg-3’ and 5’-gctccagtctcgctgaagaagg-3’,

*tsu*: 5’-tgtgttggacattgacaa-3’ and 5’-cttcgccttttccttcag-3’,

*rl*: 5’-atatcgccctttgaacac-3’ and 5’-ctctcatttggtctatgctat-3’,

*cno*: 5’-tgttccatgtgcggagacg-3’ and 5’-gacagagccaggttgtagcg-3’,

*dbo*: 5’-atccgaaggtcacgctcact-3’ and 5’-agcctcatccaccaaatccc-3’,

*kel*: 5’-acagcaagcacagccacaac-3’ and 5’-atcctcccgactccttagcg-3’,

*l(2)gl*: 5’-caggaccaaccagagcaatc -3’ and 5’-agactgtactggcatttgt-3’,

*Nek2*: 5’- cggtttgaggagccctacatc-3’ and 5’-ggaatccgactgaactcaccct-3’,

*RhoGEF2*: 5’-aggaaaccctatggatgac-3’ and 5’-ccgatccctatgaccactaa-3’,

*sano*: 5’-agcattccgcaaccactt-3’ and 5’-agcataattctgggcgtagc-3’,

*yki*: 5’-ggcgatacatcactcccg-3’ and 5’-gatcctcccactgcgtagatt-3’,

*dlg1*: 5’-cagtaccgcccagaggagt-3’ and 5’-gatccaatgtcgattgcttatc-3’,

*dlg1* for detecting aberrant transcripts: 5’-gcccgaaggctgtgccaaac-3’ and

5’-tctgctgcggctgctgatt-3’,

*dsh*: 5’-tggctaaagataacgattcc-3’ and 5’-ctgaacatcctccacattc-3’,

*wg*: 5’-atctgcctgatggccctgtg-3’ and 5’-cctggtggttcgcttgtgga-3’.
